# COVID‐19‐Related Croup With Acute Respiratory Failure

**DOI:** 10.1002/kjm2.70040

**Published:** 2025-05-15

**Authors:** Chi‐Kai Hsu, Tang‐Hsu Hsieh, Yi‐Ching Liu, I‐Chen Chen

**Affiliations:** ^1^ School of Medicine College of Medicine, Kaohsiung Medical University Kaohsiung Taiwan; ^2^ Department of Pediatrics Kaohsiung Medical University Hospital Kaohsiung Taiwan; ^3^ Department of Pediatrics School of Medicine, College of Medicine, Kaohsiung Medical University Kaohsiung Taiwan; ^4^ Department of Pediatrics Kaohsiung Medical University Gangshan Hospital Kaohsiung Taiwan

Croup is generally a self‐limiting disease and rarely requires respiratory support [1]. However, recent studies have suggested a possible association between SARS‐CoV‐2 and more severe clinical presentations, particularly during the Omicron wave of the coronavirus 2019 (COVID‐19) pandemic [1]. In this report, we share our experience managing a case of COVID‐19‐related croup with respiratory failure, and provide a review of the literature.

An 8‐month‐old boy with no notable medical history initially presented with fever, rhinorrhea, cough, and a positive SARS‐CoV‐2 nasopharyngeal rapid antigen test. He was admitted for dehydration and inadequate oral intake. On the third day of hospitalization, he developed a barking cough, with tachypnea (40–50 breaths/min) and an oxygen saturation (SpO_2_) level of 90%. Physical examination revealed moderate chest wall retraction (two points), stridor at rest (two points), significant cyanosis upon agitation (four points) and decreased breath sounds in auscultation (one point), with the Westley croup score being 9. High‐flow nasal cannula therapy was initiated at 25% FiO_2_ with a flow rate of 20 L/min, and his condition stabilized. Intravenous dexamethasone (0.6 mg/kg) and nebulized epinephrine were also administered, while routine laboratory tests and chest radiography were unremarkable.

Nevertheless, his symptoms deteriorated 5 h later, with cyanosis and SpO_2_ dropping to 85%, and his condition failed to improve despite titrating up the flow rate. He then underwent intubation and was transferred to our pediatric intensive care unit (PICU). In the PICU, he continuously received dexamethasone (0.25 mg/kg/day), remdesivir (5 mg/kg/day), nebulized epinephrine, and empirical antibiotics with amoxicillin (75 mg/kg/day). Following these interventions, he gradually improved, was successfully extubated on the sixth day of PICU admission, then discharged on the eleventh day without major complications.

Recent studies have reported increased severity of COVID‐19‐related croup [1]. One observational analysis indicated elevated risks of hospitalization, intensive care unit admission, and receiving respiratory support among omicron croup patients [1]. To our knowledge, only four cases of COVID‐19‐related croup requiring intubation have been reported in the literature [2, 3, 4]. We compared our case with the others, including clinical presentations and treatment modalities, as discussed below and shown in Table 1. Among the reported cases in Table 1, rare causes of COVID‐19‐related croup leading to acute respiratory failure include the combination of multisystem inflammatory syndrome and bacterial tracheitis [2, 3].

**TABLE 1:** Comparison of our case with other reported severe COVID‐19‐related croup cases.

Patient	Age	Gender	Pathogen (variants)	Westley score	Corticosteroid does	NRE dose	Antiviral agents	ICU time	Intubation time	LOS	Reference
Our case	8 months	M	SARS‐CoV‐2	9	2 IV DXE (0.25 mg/kg)	Yes	Remdesivir (5 mg/kg/day)	9 days	6 days	11 days	
1	18 months	N/A	SARS‐CoV‐2^a^	N/A	1 IV DXE followed by methylprednisolone (1.25 mg/kg/days)	Yes	N/A	N/A	7 days	N/A	2
2	3 years	M	SARS‐CoV‐2^b^	N/A	Yes (unknown dose)	Yes	Kaletra (ritonavir/lopinavir) (12 mg/kg/12 h)	N/A	17 days	N/A	3
3	9 months	F	SARS‐CoV‐2 (BA.2 variant)	16	IV DXE (0.5 mg/kg)	Yes	N/A	N/A	4 days	10 days	4
4	11 months	M	SARS‐CoV‐2 (BA.3 variant)	12	IV DXE (0.25 mg/kg)	Yes	N/A	N/A	N/A	9 days	4

Abbreviations: DXE, dexamethasone; F, female; ICU, intense care unit; IV, intravenous; LOS, length of stay; M, male; N/A, not available; NRE, nebulized racemic epinephrine.

^a^ Combined with multisystem inflammatory syndrome.

^b^ Combined with bacterial tracheitis ( *Staphylococcus aureus* ).

The exact mechanism by which SARS‐CoV‐2 leads to more severe croup is still not fully understood. Ex vivo studies demonstrate that the Omicron variant replicates more rapidly in the bronchus and less efficiently in the lung compared to earlier strains, partly due to its preference for the endocytic pathway, which implies a possible faster croup progression [5].

While existing management protocols for croup remain applicable to COVID‐19 cases, further research is needed to validate this approach [1]. In our case, remdesivir was administered; previous reports have also noted the use of Kaletra (ritonavir/lopinavir) in similar scenarios [3]. Although there are currently no established guidelines for the use of antiviral agents, a prior review has recommended them in severe cases or when disease progression is concerning [1].

In conclusion, due to the ongoing COVID‐19 pandemic, physicians should remain vigilant regarding the early recognition and aggressive management of croup, including considering the use of antiviral medications, particularly in severe cases.

## Conflicts of Interest

The authors declare no conflicts of interest.

## Data Availability

The data that support the findings of this study are available on request from the corresponding author. The data are not publicly available due to privacy or ethical restrictions.

## References

[1] K. L. Hon , Y. W. Tan , K. K. Y. Leung , et al., “COVID‐19 Associated Croup,” Current Pediatric Reviews 20 (2024): 453–457. 37461365 10.2174/1573396320666230718100609

[2] C. C. Lim , J. Saniasiaya , and J. Kulasegarah , “Croup and COVID‐19 in a Child: A Case Report and Literature Review,” BML Case Reports 14 (2021): e244769. 10.1136/bcr-2021-244769 PMC8442056 34521741

[3] M. Kamali Aghdam , H. Shabani Mirzaee , and K. Eftekhari , “Croup Is One of the Clinical Manifestations of Novel Coronavirus in Children,” Case Reports in Pulmonology 2021 (2021): 8877182. 33747592 10.1155/2021/8877182 PMC7943265

[4] S. Park , J. You , J. Lee , and E. Park , “Two Case Reports of Life‐Threatening Croup Caused by the SARS‐CoV‐2 Omicron BA.2 Variant in Pediatric Patients,” Journal of Korean Medical Science 37 (2022): e192. 35726145 10.3346/jkms.2022.37.e192 PMC9247726

[5] K. P. Y. Hui , J. C. W. Ho , M. C. Cheung , et al., “SARS‐CoV‐2 Omicron Variant Replication in Human Bronchus and Lung Ex Vivo,” Nature 603, no. 7902 (March 2022): 715–720. 35104836 10.1038/s41586-022-04479-6

